# Preparing the Degradable, Flame-Retardant and Low Dielectric Constant Nanocomposites for Flexible and Miniaturized Electronics with Poly(lactic acid), Nano ZIF-8@GO and Resorcinol Di(phenyl phosphate)

**DOI:** 10.3390/ma11091756

**Published:** 2018-09-18

**Authors:** Mi Zhang, Yu Gao, Yixing Zhan, Xiaoqing Ding, Ming Wang, Xinlong Wang

**Affiliations:** School of Chemical-Engineering, Nanjing University of Science & Technology, Nanjing 210094, China; 18120166132@163.com (M.Z.); gaoyu@njust.edu.cn (Y.G.); 18751851021@163.com (Y.Z.); ding1771556282@163.com (X.D.); m18751966157@163.com (M.W.)

**Keywords:** ZIF-8@GO hybrids, PLA, dielectric constant, flame-retardant, flexible

## Abstract

Degradable, flame retardant, and flexible nanocomposite films with low dielectric constant were prepared with poly (lactic acid) (PLA), nano ZIF-8@GO, and degradable flame-retardant resorcinol di(phenyl phosphate) (RDP). The SEM results of the fractured surfaces indicated that ZIF-8@GO and RDP were dispersed uniformly in the PLA matrix. The prepared films had good mechanical properties and the tensile strength of the film with 1.5 wt% of ZIF-8@GO was increased to 48.2 MPa, compared with 38.5 MPa of pure PLA. Meanwhile, the nanocomposite films were flexible due to the toughing effect of RDP. Moreover, above 27.0% of limited oxygen index (LOI) and a VTM-0 rating were achieved for the nanocomposite films. The effects of nano ZIF-8@GO hybrids and RDP on the dielectric properties were investigated, and the results showed that ZIF-8@GO and RDP were beneficial in reducing the dielectric constant and dielectric loss of the nanocomposites.

## 1. Introduction

In recent years, electronic products such as mobile phones have been updated faster, which results in a large volume of e-waste and poses a risk to the sustainable environment [1]. It is estimated that globally, about 20–50 million tons of waste electronic equipment is discarded annually [2]. Green electronic products made of environmentally friendly and disposable materials, such as degradable polymers, are ideal candidates to resolve the pollution from electronic waste [3,4].

Owing to the miniaturization, portability, and flexibility requirements of electronic products, polymeric materials with low dielectric constant and flexibility are getting more attention in integrated circuit devices as interlayer dielectrics [5,6,7]. By reducing the dielectric constant of the dielectric material used in the integrated circuit, the leakage current of the integrated circuit, the capacitance effect between the wires, as well as the heating of the integrated circuit can be reduced efficiently [8]. Another important problem when thinking of the application of polymer-based low dielectric materials, is that most polymeric materials are easy to burn [9]. To use electronic equipment safely, it is essential that the polymer-based dielectric layer should have flame retardant properties.

Nanocomposites exhibit improved physical properties, compared to those of the unfilled polymer matrix, i.e., Mechanical strength [10,11], dielectric properties [12,13], thermal properties [14], and fire resistance [15,16], etc. Oliviero et al. [17] studied the dielectric properties of sustainable nanocomposites, based on zein protein and lignin for biodegradable insulators. Santanu Singha et al. [18] investigated the effects of nano ZnO on the dielectric properties of epoxy nanocomposites and found that the volume fraction and nature of the interfaces in most of the composites, influence the dielectric properties of the nanocomposites. Rao [19] investigated the structural and electrical properties of novel polyvinyl alcohol (PVA)-CuO nanocomposite films, and the results showed that the dielectric constant was reduced with an increase in both frequency and CuO concentration; and the dielectric loss was increased with increase in frequency and decreased with increase in CuO concentration. Regarding the flame retarded properties of nanocomposites, Hapuarachchi [20] developed the poly(lactic acid) (PLA) nanocomposites with improved flame retardancy, utilizing the unique properties of sepiolite nanoclay and multi-7walled nanotubes. Li groups [21] developed the core-shell nanostructured MWCNT-DOPO-OH through a three-step process and added them to aluminum hypophosphite/poly(lactic acid) (AHP/PLA) flame retardant systems, to improve both flame retardancy and mechanical properties. The results indicated that the PLA nanocomposites with 1 wt% MWCNT-DOPO-OH and 14 wt% AHP, achieved a UL 94 V-0 rating and limiting oxygen index (LOI) value of 28.6%. Ye et al. [22] reported that the addition of organically modified montmorillonite (OMMT) and aluminium diethylphosphinate (AlPi) into the PLA matrix could promote char-forming and suppress the melt dripping. However, few papers have reported on the preparation of degradable nanocomposites with good flame retardancy and low dielectric constant, simultaneously.

**Scheme 1:**
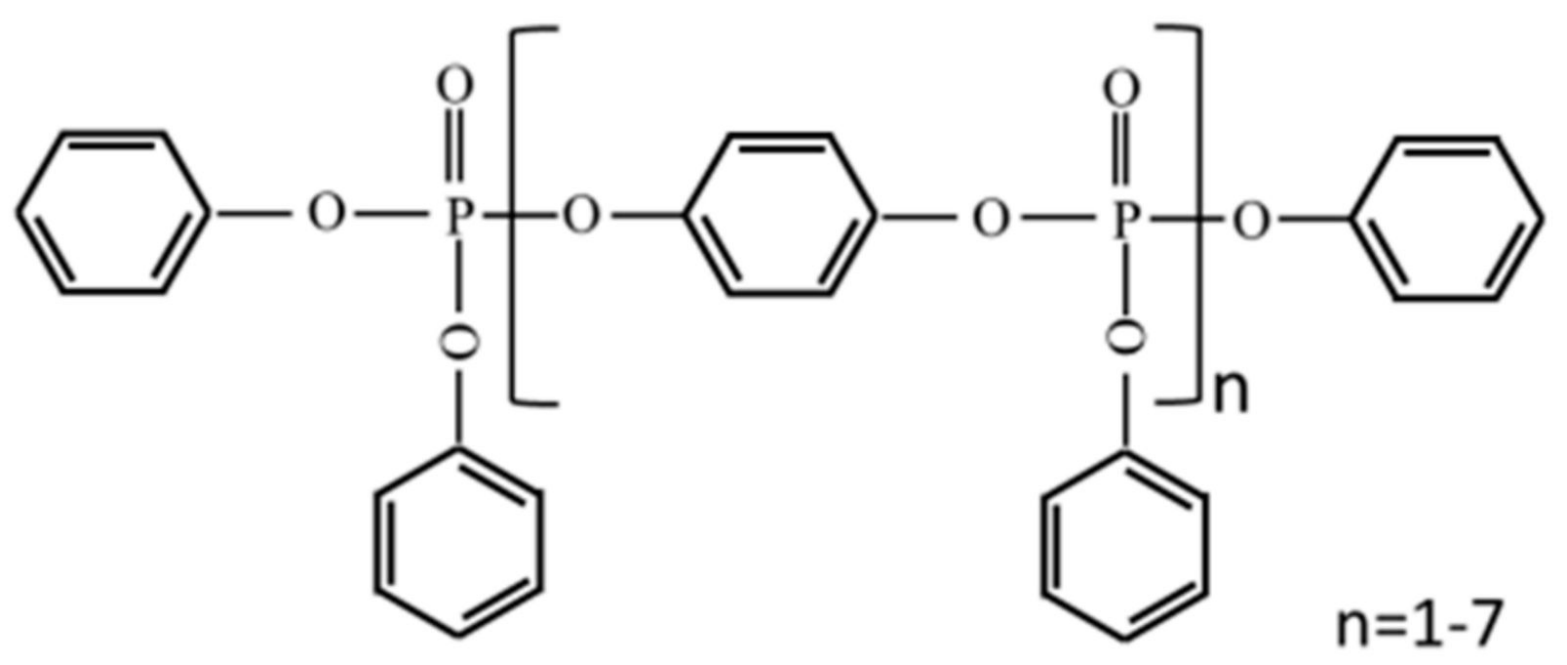
The chemical formula of resorcinol di(phenyl phosphate) (RDP).

In this paper, PLA, nano-ZIF-8@GO hybrids of nano zeolite imidazole frameworks (nano-ZIF-8) and graphene oxide (GO), and resorcinol di(phenyl phosphate) (RDP) were used to prepare the nanocomposites. PLA, a kind of biodegradable polyester produced from renewable resources, is now increasingly viewed as a valuable biosourced polymer alternative in applications, such as electronics [23,24]. The hybrids combine the unique advantage of metal–organic frameworks (MOFs) and GO, such as crystalline and highly ordered structures, ultrahigh porosity and large surface area, two-dimensional structure with rich carboxyl, hydroxyl, and epoxy groups [25]. The flame-retardant RDP is a biodegradable polymeric compound with good thermal stability, as shown in Scheme 1. The effects of nano ZIF-8@GO hybrids and RDP on dielectric behavior, mechanical properties, and flame retardancy of the PLA nanocomposite films have been systematically studied.

## 2. Experimental

### 2.1. Materials

Poly (lactic acid) (PLA 290, *M*_W_ = 50,000−60,000, D = 1.4–1.5, content of D-lactide = 0.5%) was obtained from Haizheng Biological Materials Co., Ltd., Taizhou, China. Resorcinol bi(diphenyl phosphate) (RDP) was provided by Wansheng New Material Co., Ltd., Linhai, China. 2-Methyl imidazole (98%) was purchased from Aladdin Industrial Corporation, Shanghai (China). Zn(NO_3_)_2_·6H_2_O was supplied by Xilong Chemical Co., Ltd., Shantou, China. Deionized water was produced in our lab. Methanol (CH_3_OH), graphite powders, phosphorus pentoxide (P_2_O_5_), potassium persulfate (K_2_S_2_O_5_), and hydrogen peroxide (H_2_O_2_) were provided by Sinopharm Chemical Reagent Co., Ltd., Shanghai, China. Concentrated sulfuric acid (H_2_SO_4_, 95~98%), Chloroform (CHCl_3_, 99%), potassium permanganate (KMnO_4_), and hydrochloric acid (HCl) were obtained from Lingfeng Chemical Reagent Co., Ltd., Shanghai, China.

### 2.2. Preparation of ZIF-8@GO

Graphene oxide (GO) was prepared with an improved Hummers method [26]; 1.602 g of 2-methyl imidazole and 1.487 g of Zn(NO_3_)_2_·6H_2_O were dissolved in 100 mL of methanol (CH_3_OH), respectively, and 0.163 g of GO was dispersed in 100 mL of CH_3_OH. Then, the GO suspension was added to the above solution with stirring. Finally, the reaction mixture was stirred for 1h at room temperature and ZIF-8@GO particles were centrifuged, washed with CH_3_OH, and then dried at 80 °C for 48 h.

### 2.3. Preparation of the Nanocomposite Films

First, the dried PLA was dissolved in 40 mL of chloroform (CHCl_3_) with magnetic stirring for 2 h. An appropriate amount of ZIF-8@GO and RDP was dispersed in 20 mL of CHCl_3_ by sonication to form a uniform dispersion solution, and then poured into the PLA solution. The mixture was stirred for 4 h and kept for 1 h. The mixed solution was then casted into a film using an automatic coater (MRXTMH250, Mingruixiang Automation Equipment Co., Ltd., Shenzhen, China). After the solvent was evaporated at room temperature, the film was dried in an oven at 50 °C for 72 h, to further remove the residual solvent. The concrete formulations of nanocomposite films are listed in Table 1.

### 2.4. Measurement and Characterization

Transmission electron microscope (TEM, JEM-2100, Tokyo, Japan) was operated to observe the morphologies of ZIF-8@GO.

X-ray diffraction (XRD, Bruker D8 Advance diffractometer, Karlsruhe, Germany) measurements were performed at 40 kV and 40 mA, with Cu K_α_ radiation (0.15418 nm).

Fourier transform infrared spectroscopy (FTIR) spectra were recorded on a FTIR-8400S spectrometer (Shimadzu, Japan), at a range of 400–4000 cm^−1^, with a resolution of 4 cm^−1^, to observe the structure of PLA nanocomposites.

Scanning electron microscope (SEM, Hitachi S-4800, Tokyo, Japan) with an Energy Dispersive Spectrometer (EDS, GENESIS 2000, EDAX, Mahwah, NJ, USA) was employed to observe the fracture morphologies of PLA nanocomposite film. All samples were coated with gold before the test.

Tensile testing measurements were performed on a CMT tensile tester (Sans, Shenzhen, China), at a rate of 10 mm/min at room temperature, referred to ISO 1184-1983 (GB 13022-1991, China). The sample values were averaged by five measurements and sample size was (150 mm ± 2 mm) × (20 mm ± 2 mm). The thickness of the sample was 0.15 mm.

Limiting oxygen index (LOI) tests were measured according to ASTM Standard D2863-97, with a JF-3 oxygen index meter from Jiangning Analysis Instrument Co., Ltd. (Nanjing, China). The tests were performed five times, and sample size was (200 mm ± 5 mm) × (50 mm ± 2 mm).

Vertical burning tests were performed according to ISO 9773 using a vertical burning tester (CZF-3, Jiangning Analytical Instrument Co., Ltd., Nanjing, China), with a standard of UL-94. The sample values were measured five times, and sample size was (200 mm ± 5 mm) × (50 mm ± 2 mm). The thickness of the sample was 0.15 mm.

Thermogravimetric analysis (TGA) was carried out from 35 °C to 600 °C using a DTG-60 thermoanalyzer instrument (Shimadzu, Tokyo, Japan), at 20 °C/min under nitrogen. The mass of the samples was kept within 3–5 mg in an alumina crucible.

The dielectrical characterization of the nanocomposites was performed using a high-frequency LCR meter (TH2816, L = 100 μH, K = 6, DZ5001, Nanjing, China), with a frequency range from 1 kHz to 150 kHz, as described in Reference [27]. In the experimental process, air was used as a reference. The permittivity (*ε_r_*) was calculated from the formula as follows:

*ε_r_* = 14.4 · (*C*_1_ − *C*_2_)* · d*/*Φ*^2^ (1)

where *C*_1_, *C*_2_ is the capacitor of air and sample (pF), and *d* is the thickness of the film (cm). *Φ* is the diameter of the film (cm) and the general value of *Φ* was 3 cm. The dielectric loss tangent (*tanδ*) was calculated from the formula as follows:

*tanδ* = *C*_1_ · (*Q*_1_ − *Q*_2_)/(*C*_1_ − *C*_2_) · *Q*_1_ · *Q*_2_ (2)

where *Q*_1_, *Q*_2_ is the quality factor of air and sample, respectively.

## 3. Results and Discussion

### 3.1. Characterization of ZIF-8@GO

**Figure 1:**
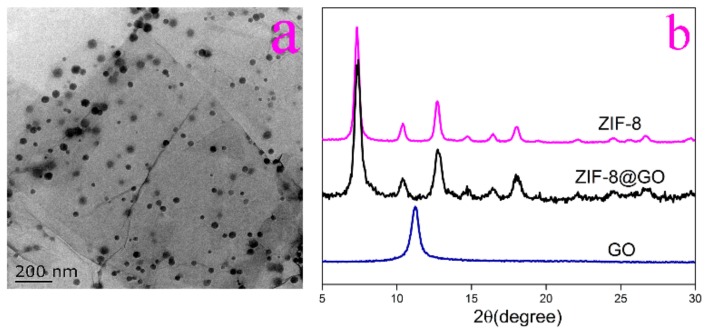
(**a**) Transmission electron microscope (TEM); (**b**) X-ray diffraction (XRD) of ZIF-8@GO.

The morphologies of ZIF-8@GO are shown in Figure 1a. It is apparent that many ZIF-8 nanoparticles were anchored on the surface of GO sheets by chelation between oxygen-containing functional groups on GO and Zn ions in ZIF-8 [28]. Figure 1b shows the XRD patterns of ZIF-8, GO, and ZIF-8@GO. The peak centered at 10.9° for GO, corresponds to the reflections of the (002) plane [29]. The XRD pattern of the synthesized ZIF-8 was similar to that reported in previous literature [30]. The XRD pattern of ZIF-8@GO showed the diffraction peaks of ZIF-8. However, the (002) reflection of GO was absent because the incorporation of ZIF-8 had destroyed the regular stack of GO. This phenomenon was inconsistent to those reported by others in References [31,32]. The XRD analysis further demonstrated that ZIF-8 nanoparticles had been effectively loaded onto the GO sheets.

### 3.2. SEM and Transparency of the Nanocomposite Films

**Figure 2:**
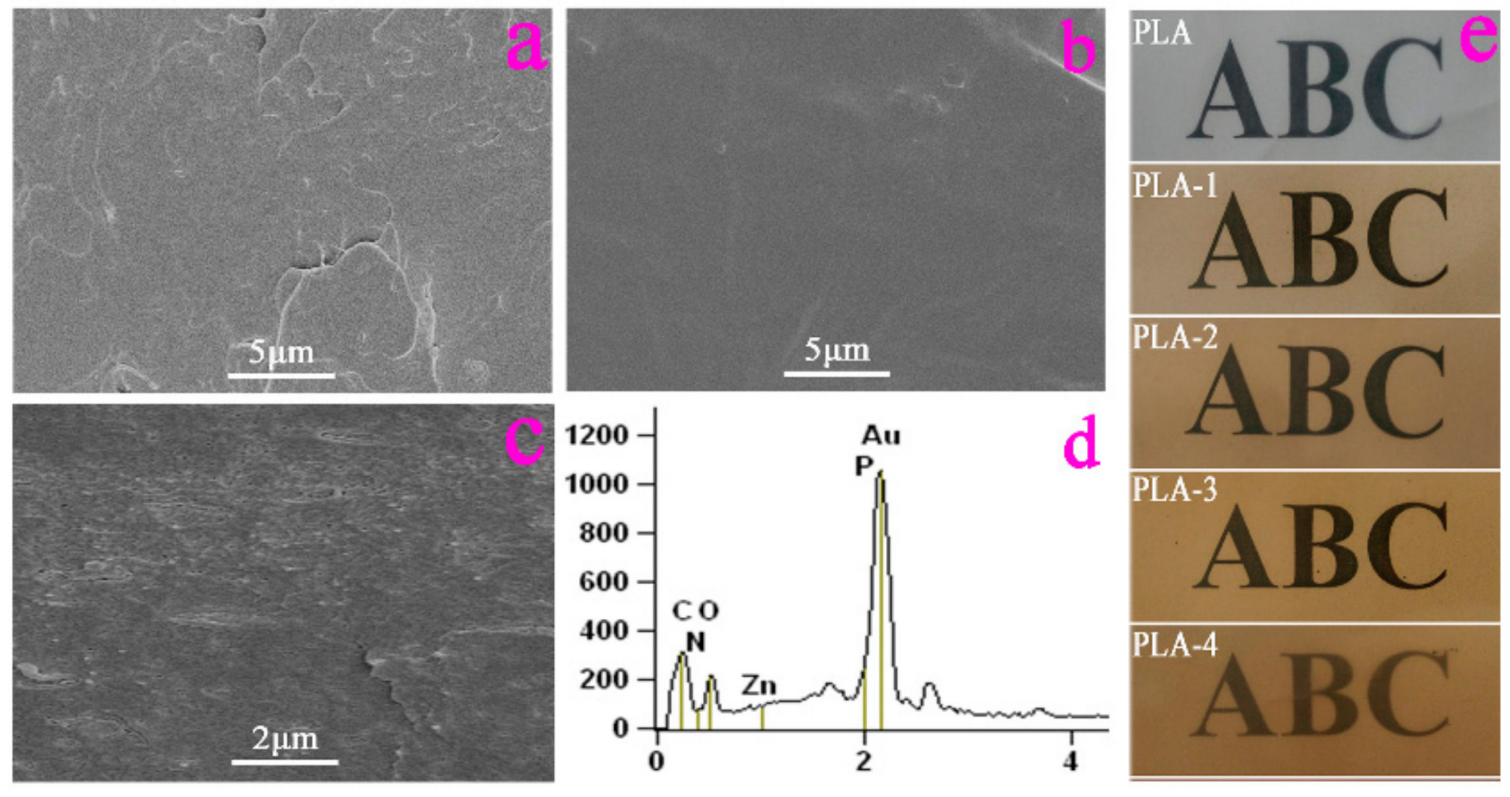
(**a**) Scanning electron microscope (SEM) of pure poly (lactic acid) (PLA); (**b**,**c**) SEM of PLA-2; (**d**) EDS of PLA-2; (**e**) Optical images of the prepared films.

Figure 2a shows the SEM image of pure PLA. As can be seen, pure PLA exhibited a fractured morphology with some cracks, which is the characteristic of rigid and fragile materials. The fractured morphology of PLA-2 with 0.9 wt% ZIF-8@GO and 9.0 wt% RDP is shown in Figure 2b,c, and the surface with no large agglomerations is observed, indicating that ZIF-8@GO and RDP are dispersed uniformly in the PLA matrix. RDP is a kind of organic polyphosphate and is miscible with PLA [33]. Regarding the good dispersion of ZIF-8@GO in PLA, the strong interactions between the organic linkers in ZIF-8 and PLA chains provide good affinity for ZIF-8@GO with PLA. Moreover, ZIF-8 nanoparticles anchored on GO sheets prevented GO from stacking together and made them disperse well in the PLA matrix. Moreover, the surface of ZIF-8@GO may be modified by the organic RDP, since many P=O and P–O–C groups in RDP can form hydrogen bonds with the –OH groups existing on the surface of GO sheets, or coordination bonds with Zn ions in ZIF-8. Thus, RDP molecules tend to be absorbed on the surface of ZIF-8@GO nanoparticles, which enhances the compatibility between ZIF-8@GO nanoparticles and the PLA matrix. The EDS of PLA-2 nanocomposite shown in Figure 2d, further proves the presence of ZIF-8@GO and RDP in the nanocomposites. In Figure 2e, all the films are found to be transparent enough to see “ABC” letters on paper beneath the films clearly, even though the content of ZIF-8@GO reaches 1.5 wt%.

### 3.3. Mechanical Properties of the Nanocomposite Films

**Figure 3:**
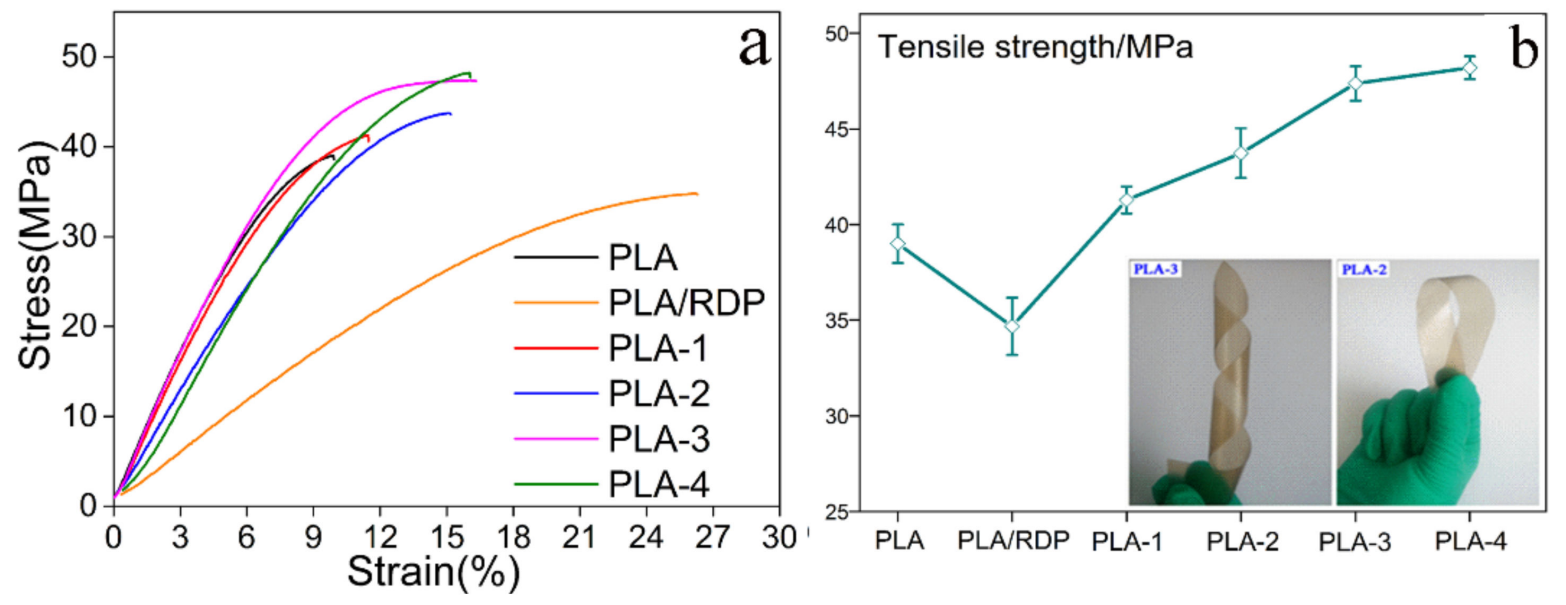
The stress-strain curves (**a**) and tensile strength (**b**) of the nanocomposite films.

**Figure 4:**
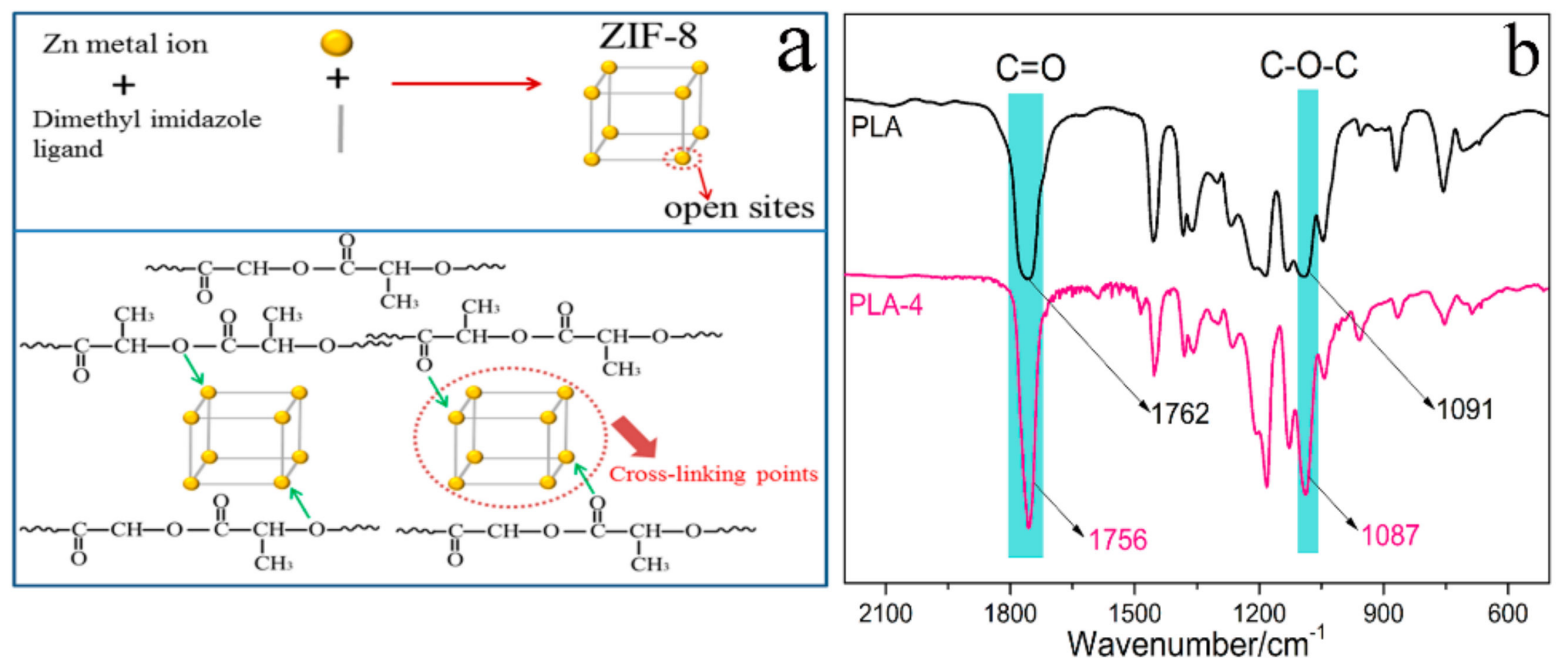
The enhancement mechanism (**a**) and Fourier transform infrared spectroscopy (FTIR) curves (**b**) the nanocomposite films.

The typical stress-strain curves for the nanocomposite films are shown in Figure 3a. The linear stress-strain behavior of films in the initial region was observed as the ‘Hookean’ region, which was due to the changes in bond angles and spacing of PLA [34]. The rest of the region was considered as the plastic region, which is attributed to the movement of the PLA chain under the external force. The related data of tensile strength from the stress-strain curve, is illustrated in Figure 3b. It is well known that PLA is a rigid thermoplastic polymer with 38.5 MPa of tensile strength; however, the tensile strength of PLA/RDP film is only 34.7 MPa, which may be ascribed to the fact that the RDP molecules inserts PLA chains and increases the spacing of PLA chains, weakening the inter-chain stress during the blending process, resulting in reduced tensile strength [35]. The nanocomposite films show a trend of increase in tensile strength, for example, when the content of ZIF-8@GO was 1.2 wt%, the tensile strength of PLA-3 was 47.4 MPa, which was an increase of 22.8% compared with the pure PLA. This may be owing to the fact that the added ZIF-8@GO particles played a reinforcing effect for PLA, where the Zn-containing groups of ZIF-8@GO could interact with the ester groups of PLA to form a partial crosslinking through coordination interaction, as exhibited in Figure 4a. In Figure 4a, the Zn-containing groups of ZIF-8@GO have open sites, as described in Reference [36], which have a strong coordination effect with C=O and C–O–C of ester groups in PLA. To support this conclusion, the FTIR curves of PLA and PLA-4 as a representative are exhibited in Figure 4b. The peak at 1761 cm^−1^ was attributed to the stretching vibration of C=O in PLA [8]. While adding ZIF-8@GO, the peak shifted to 1756 cm^−1^ for PLA-4. Similarly, the peak at 1091 cm^−1^, corresponding to the C–O–C vibration in PLA shifts the lower wavenumber. The shifts of these peaks could be due to the strong interaction between PLA and ZIF-8@GO. Moreover, RDP, as a compatibility agent, promotes the uniform distribution of ZIF-8@GO in PLA, which is beneficial to the improvement of mechanical properties [37,38]. The digital photo for the PLA films in Figure 3b, shows that the prepared nanocomposite films are so flexible, which could be due to plasticity of RDP for PLA, as well as the toughing effects of ZIF-8@GO in PLA [39]. The elongations at break of PLA-1, PLA-2, and PLA-3 are about 11.48%, 15.17%, and 16.33%, respectively, compared with 9.92% for PLA.

### 3.4. Flame Retardant Properties of the Nanocomposite Films

**Figure 5:**
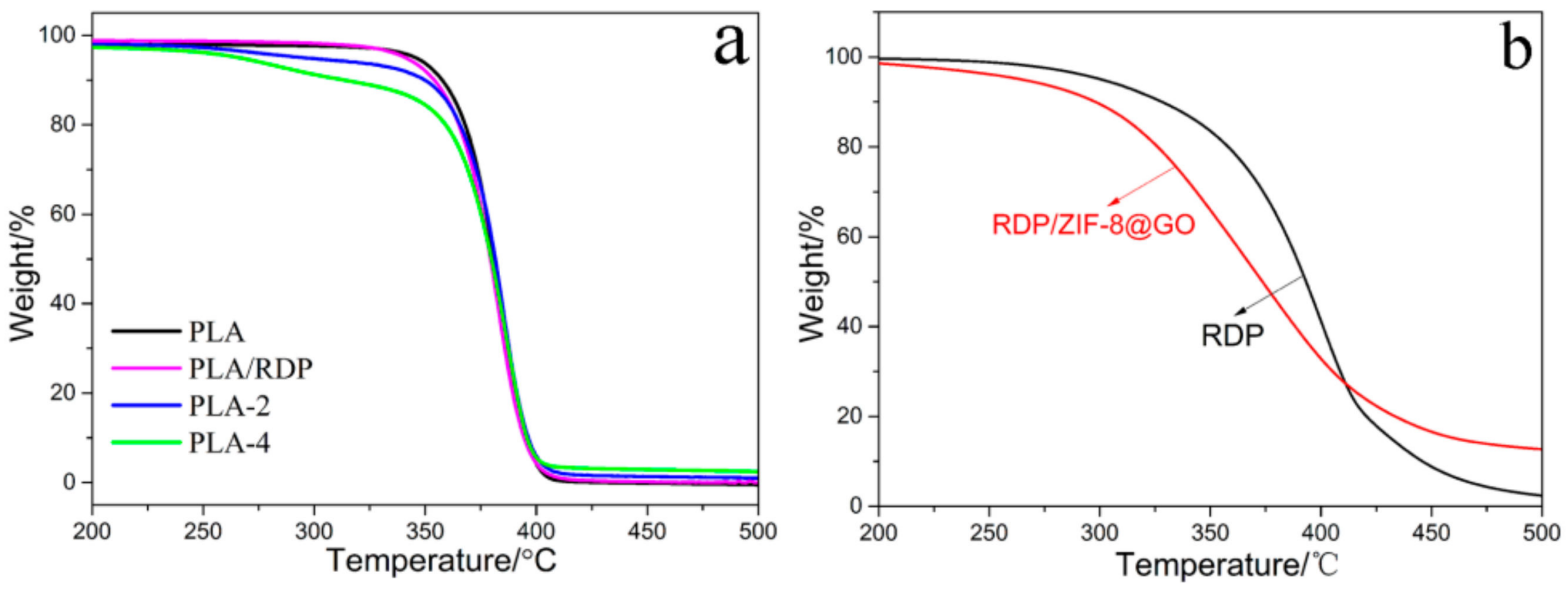
TG curves of PLA nanocomposite films (**a**); RDP and RDP/ZIF-8@GO blend (**b**).

**Figure 6:**
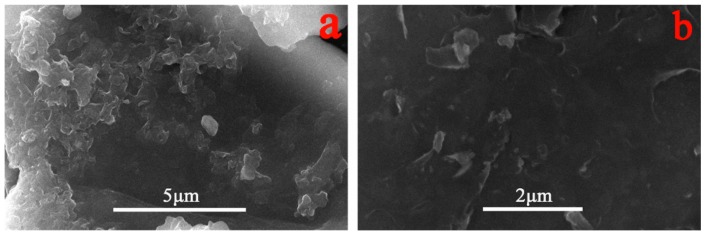
SEM images of PLA-2 nanocomposite films after combustion under different resolutions. (**a**) 5 μm; (**b**) 2 μm.

Table 1 shows the results of the combustion performance for the nanocomposite films, including the limit oxygen index (LOI) and vertical burning rating. The LOI is defined as the minimum oxygen concentration required for combustion under the oxygen-nitrogen atmosphere. It is generally believed that pure PLA has a poor flame-retardance property, as its LOI is only 21.0%. In addition, PLA burns vigorously with serious droplet and no residues, which will lead to the spread of flames and secondary combustion hazards, as reported previously in Reference [40]. The incorporation of RDP can remarkably improve the flame retardancy of PLA, with a LOI of 31.0% and VTM-0 rating. With respect to the flame-retardant mechanism, the organophosphorus flame retardants often play an effective role on flame retardancy, both in condensed and gas phase. In condensed phase, they decompose into phosphoric acid and pyrophosphate, which can catalyze charring process to form the char layer on polymer. In gas phase, they can react with the degrading polymer to quench radicals or produce PO· radicals, which can react with the H· or OH·, and thus act as a fire retardant. As shown in Figure 5a, the amounts of char residues of the nanocomposites are poor, suggesting RDP cannot promote the carbonization of PLA. Therefore, we speculate that the gas phase mechanism is mainly responsible for the improvement of flame retardancy, for the prepared nanocomposites. Fang has proved the statement that some organophosphorus flame retardants are mainly active in the gas phase and not in the condensed phase using TG-FTIR [41]. The addition of ZIF-8@GO into the PLA films results in a slightly lower LOI, but remains in high values. For instance, when the ZIF-8@GO content is 1.5 wt%, the LOI of PLA-4 is 27.0%. The reduction in flame retardance may be attributed to ZIF-8@GO nanoparticles, which catalyzes the decomposition of RDP at low temperature regions, as shown in Figure 5. In Figure 5a, the initial decomposition temperatures of PLA and PLA/RDP are about 345.5 °C and 340.9 °C. While adding the RDP and ZIF-8@GO, the initial decomposition temperatures of PLA-2 and PLA-4 nanocomposites were about 295.1 °C and 264.4 °C, which were lower than 340.9 °C for PLA/RDP. Moreover, the TG curves of RDP and the RDP/ZIF-8@GO blend are obtained in Figure 5b. The initial decomposition temperatures of RDP and RDP/ZIF-8@GO blend were 300.4 °C and 265.0 °C, respectively. The RDP/ZIF-8@GO blend decomposed earlier than that of RDP, which proved the catalytic effect of ZIF-8@GO further [42,43]. The decomposition of RDP at low temperature impairs the effect of flame retardancy of RDP. The SEM of char residue after combustion of the nanocomposites (PLA-2 film), is shown in Figure 6. Although this char layer is continuous and beneficial to improvement of flame retardance of the PLA nanocomposites, the amount of char residue after combustion of the nanocomposites is so poor that it cannot improve flame retardancy effectively in condensed phase.

### 3.5. Dielectric Properties of the Nanocomposite Films

**Figure 7:**
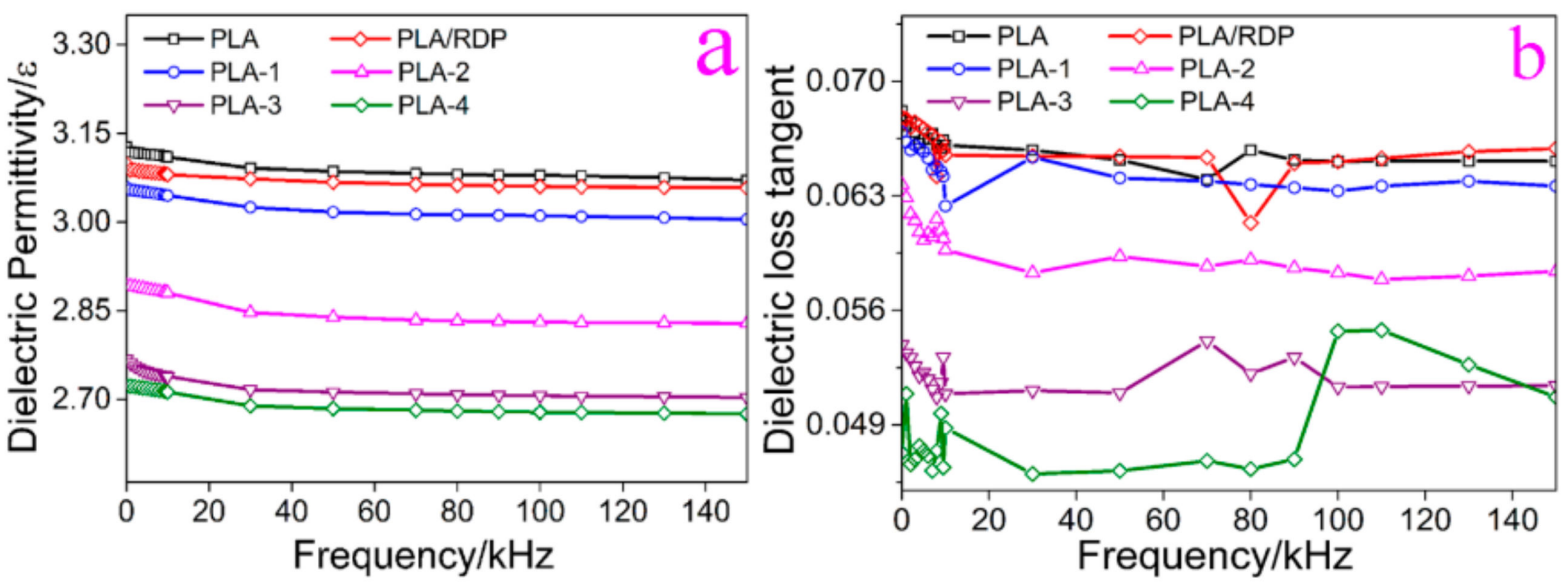
Dielectric permittivity (**a**) and dielectric loss tangent (**b**) of the films.

The dielectric constant and dielectric loss tangent are the most important parameters for dielectric materials. The dielectric constant and dielectric loss tangent of the nanocomposite films from 1 kHz to 150 kHz have been tested, and the results are shown in Figure 7. As shown in Figure 7a, the dielectric constant show low-frequency dependence within the measuring frequency. When the frequency is less than 30 kHz, the dielectric constant decreases rapidly with the frequency. This is because the dielectric constant of the PLA films, depends on their abilities to polarize at a given frequency. There are four possible polarizations that could contribute to the dielectric behavior: Electronic, ionic, dipolar, and interfacial polarization [44]. At low frequencies, all four types of polarization have enough time to occur, which is helpful to reduce the dielectric constant. As the frequency increases, the contributions of interfacial, dipolar, and ionic polarizations do not have enough time to adjust to the change of frequency and become ineffective, only the electronic polarization still plays a role in the dielectric constant [8,45]. The decrease of dielectric permittivity with increasing frequency is due to the relaxation behaviors [46]. Figure 7b shows the dielectric loss tangent for the films from 1 kHz to 150 kHz. In the range of the discussed frequency, the change of dielectric loss is not obvious. It is seen that the values of all the films are in a range from 0.045 to 0.070.

**Figure 8:**
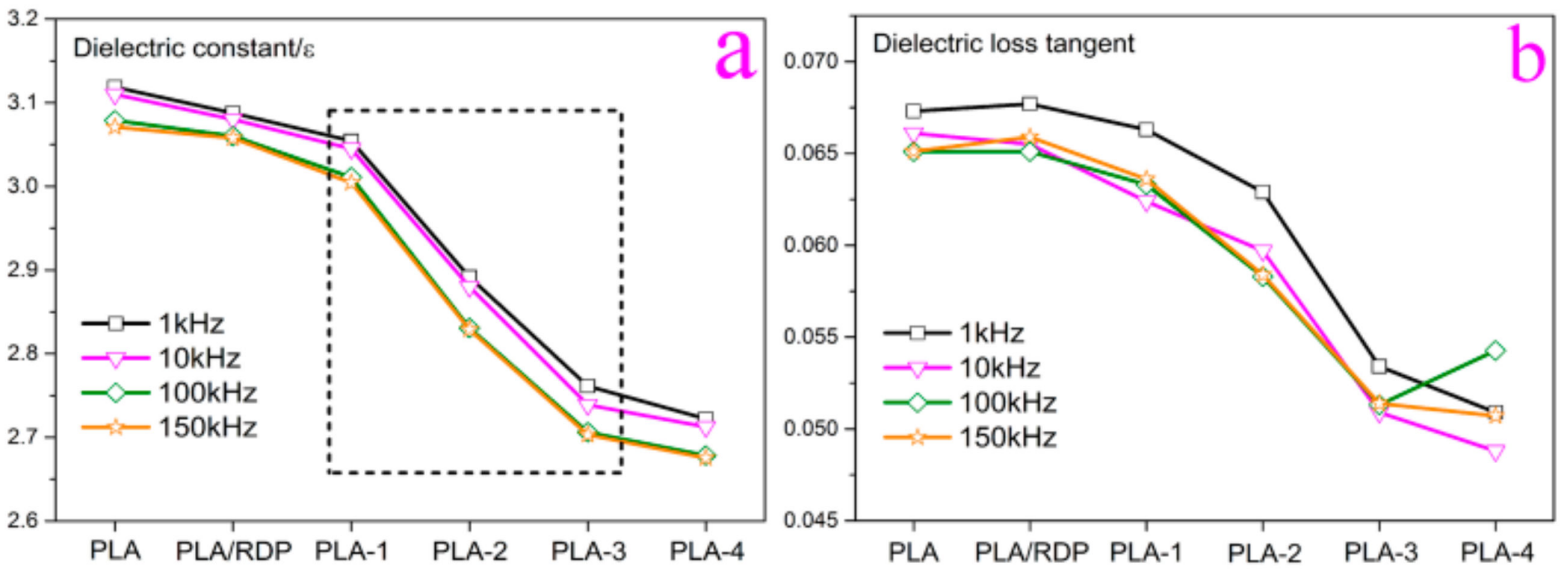
Dielectric permittivity (**a**) and dielectric loss tangent (**b**) with the content of ZIF-8@GO.

**Figure 9:**
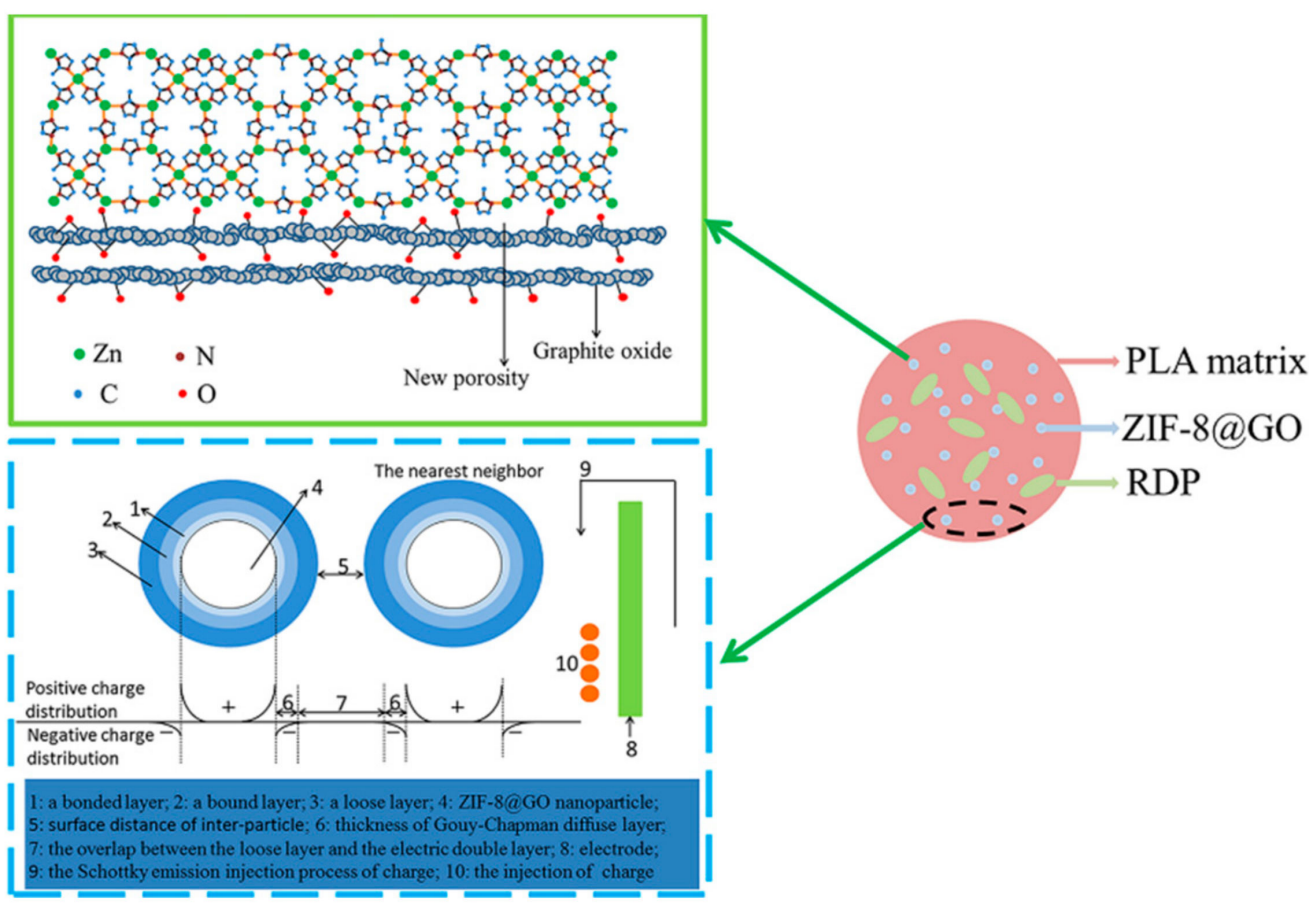
Mechanism of the dielectric permittivity reduction for the films. (**a**) Nanoparticle effect; (**b**) Interface effect [57].

The effects of ZIF-8@GO and RDP on the dielectric properties of the nanocomposites are shown in Figure 8. While only adding RDP, the dielectric constant decreases slightly compared with the pure PLA, which may be ascribed to the presence of several benzene rings in RDP, because it is well-known that the benzene ring has a very low dipole moment; for example, the dielectric constant of polystyrene is about 2.4 at 10^3^ kHz [47,48]. With addition of ZIF-8@GO, the dielectric constant had a rapid decline and achieved the lowest value at 1.5 wt% of ZIF-8@GO. For example, the dielectric constant of PLA-4 reached 2.61, compared with 3.1 for pure PLA at 10 kHz. The decrease of the dielectric constant is attributed to the good dispersion of fillers [49], nanoparticle effect of ZIF-8@GO, as well as the interface effect [46], as shown in Figure 9. ZIF-8 with a nano porous structure increased the porosity density in the PLA matrix, thereby reducing the polarization molecular density [50]. GO, with the special two-dimensional planar structure, lessened the efficiency of molecular piling and increased the free volume of the PLA, thereby diluting the density of polarized molecules in the PLA matrix [51]. The presence of GO also created a significant enhancement in porosity, owing to the formation of new pores at the interface of GO and ZIF-8 particles, as exhibited in Figure 9 [52]. Furthermore, as shown in Figure 9, the PLA molecules and the ZIF-8@GO nanoparticles in both the first layer and the second layer of the interface multi-core region have a strong interaction, which restricts end-chain or side-chain movement. The interaction has a profound effect on the dielectric behavior at low frequency [53,54]. However, the collaborative effect between ZIF-8@GO and RDP on the dielectric constant needs to be further investigated. Figure 8b shows the change of the dielectric loss tangent, as a function of the addition of ZIF-8@GO. It was observed that the dielectric loss decreases with an increase in the ZIF-8@GO content. The phenomenon could be explained as the Coulomb blockade effect of ZIF-8@GO. The ZIF-8@GO nanoparticles could cause a high charging energy for the tunneling electrons and inhibit the charge transfer through the whole system from migrating directionally, reducing the conduction loss which represents the flow of charge through the dielectric materials [55,56].

## 4. Conclusions

In conclusion, ZIF-8@GO hybrids were synthesized and characterized by TEM and XRD. The ZIF-8@GO particles were homogenously dispersed in the PLA matrix due to the action of RDP, as well as the interfacial interaction between PLA and ZIF-8@GO. The tensile strength was improved, compared with pure PLA, due to the reinforcing effect of ZIF-8@GO. The nanocomposite films showed good flame retardance. The LOI of the PLA-2 film was 28.5% and a VTM-0 rating was obtained. The dielectric constant of PLA films was decreased, owing to the nanoparticle and interface effects. While adding 9.0 wt% RDP and 1.5 wt% ZIF-8@GO, the dielectric constant of PLA-4 reached 2.61, compared to about 3.30 and 4.40 for PI and EP at 1 kHz frequency, respectively. Moreover, the dielectric loss tangent had a general trend of slightly decreasing with the addition of ZIF-8@GO, which could be explained as the Coulomb blockade effect of ZIF-8@GO.

## Figures and Tables

**Table 1:** Formulations and combustion tests of nanocomposite films.

Samples	PLA (wt%)	RDP (wt%)	ZIF-8@GO (wt%)	LOI (%)	Rating
PLA	100	0	0	21.0 ± 0.2	NR ^b^
PLA/RDP	91.0	9.0	0	30.0 ± 0.3	VTM-0
PLA-1	90.4	9.0	0.6	29.3 ± 0.5	VTM-0
PLA-2	90.1	9.0	0.9	28.5 ± 0.2	VTM-0
PLA-3	89.8	9.0	1.2	27.8 ± 0.6	VTM-0
PLA-4	89.5	9.0	1.5	27.0 ± 0.4	VTM-2

NR ^b^: no rating.
